# Integration of biochar and *Bradyrhizobium japonicum* modulates soil physicochemical properties and microbial community in soybean fields

**DOI:** 10.3389/fpls.2025.1723509

**Published:** 2025-12-18

**Authors:** Sikandar Aziz, Yilong Bi, Faizur Rehman, Muhammad Ibrahim, Syed Majid Rasheed, Shahid Khan, Chengyu Wang, Shuxia Liu

**Affiliations:** 1 College of Resources and Environment, Jilin Agricultural University, Changchun, China; 2 College of Agronomy, Jilin Agricultural University, Changchun, China; 3 College of Plant Protection, Jilin Agricultural University, Changchun, China; 4 Department of Plant Breeding and Genetics, The University of Agriculture Swat, Swat, Pakistan; 5 Faculty of Agriculture Sciences, Universidade Federal da Grande Dourados (UFGD), Dourados, MS, Brazil

**Keywords:** biofertilizers, microbial inoculants, nitrogen fixation, rhizosphere, soybean yield, soil fertility

## Abstract

Declining soil fertility and reduced microbial diversity due to intensive farming threaten sustainable agriculture. This study aimed to assess the effects of *B. japonicum* inoculation and biochar amendment, applied individually and in combination, on soil properties, microbial communities, and soybean yield under field conditions in Jilin Province, China. A field experiment was established with four treatments: control (CK), *B. japonicum* alone (RH), biochar alone (CK2), and their combination (RHB). Observations were recorded at flowering (FS), seed-filling (SFS), and harvesting (HS) stages. Soil physicochemical properties, microbial diversity, and soybean yield were evaluated to determine treatment responses. The RHB treatment significantly improved soil fertility. Soil pH increased from 5.77 in CK to 6.20 in RHB, total nitrogen rose from 0.12% to 0.19%, and available phosphorus increased from 32.4 to 45.3 mg/kg. Available potassium and soil organic matter increased by 18.2% and 27.7%, respectively. Soybean yield was highest in RHB (3798 kg/ha) compared to CK (3158 kg/ha) and correlated strongly with total nitrogen (TN) (*r* = 0.84), nitrate nitrogen (*r* = 0.75), and available nitrogen (*r* = 0.67). Microbial analysis revealed enrichment of beneficial genera, including Pseudomonas and Beauveria, along with higher populations of nitrogen-cycling bacteria and mycorrhizal fungi, thereby enhancing nutrient cycling. *B. japonicum* inoculation combined with biochar significantly enhanced soil fertility, improved microbial diversity, and increased soybean yield, offering a sustainable strategy to strengthen soil health and productivity in intensive farming systems.

## Graphical Abstract

**Figure:**
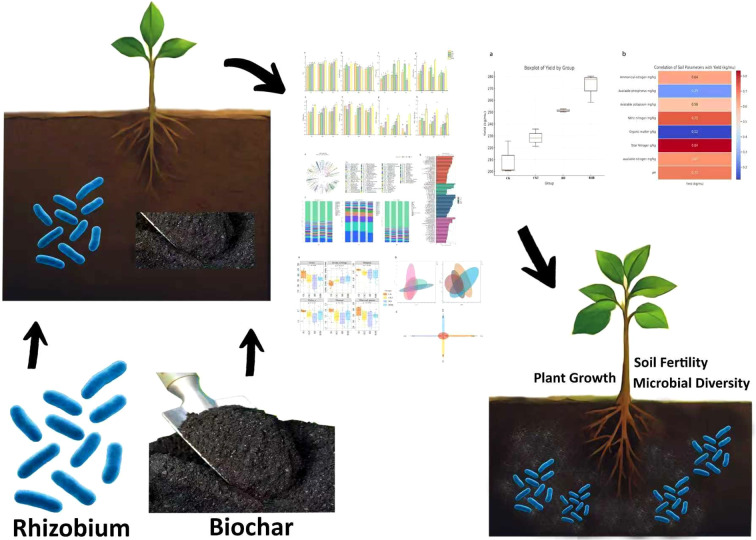

## 1 Introduction

Soybean, as a widely cultivated leguminous crop, is integral to sustainable agricultural systems due to its ability to form a symbiotic relationship with *B. japonicum*, which enables nitrogen fixation (Goyal et al., 2021; Otaiku et al., 2022). This process is crucial in replenishing soil nitrogen and minimizing the need for synthetic fertilizers, a key aspect of environmentally sustainable farming systems (Abdul-Aziz et al., 2025; Kebede, 2021). The increasing global demand for food and the simultaneous environmental challenges call for innovative, eco-friendly strategies to maintain soil health, boost agricultural productivity, and reduce reliance on chemical inputs (Islam et al., 2022; Zou et al., 2024). In this context, incorporating biological amendments, such as *B. japonicum* inoculation and biochar, represents a promising avenue to enhance soil fertility, promote beneficial microbial diversity, and improve crop yields.

As the foundation of agriculture for premium and high-yield vegetables, the development and use of chemical fertilizers are essential to address human food security and ensure a sufficient supply of horticulture products. However, growers are at risk of overusing chemical fertilizers in vegetable production because they lack access to optimal cultivation practices and are overly focused on high yield. The sustainability of agriculture is adversely affected by such excessive fertilizer application. This leads to low fertilizer efficiency and poor production benefits, resource waste and product quality losses, and other environmental issues (Lyu et al., 2023).

*B. japonicum* inoculation is a well-established practice for improving nitrogen availability in leguminous crops like soybean. By facilitating biological nitrogen fixation (BNF), *B. japonicum* enhances soil nutrient cycling, improves soil structure, and reduces the dependence on synthetic nitrogen fertilizers, which are not only costly but also contribute to soil acidification, greenhouse gas emissions, and nutrient imbalances (Huang, 2024; Otaiku et al., 2022; Sridhar et al., 2023). By forming root nodules, *B. japonicum* converts atmospheric nitrogen into a bioavailable form, significantly increasing the nutrient availability to the plant (Abd-Alla et al., 2023; Maitra et al., 2023). This process contributes to improved crop resilience and productivity, particularly in nitrogen-limited soils, and has been widely used to enhance the growth of legumes such as *Glycine max*, *Phaseolus vulgaris*, and *Cicer arietinum* (Bamdad et al., 2022; Midler, 2022; Mishra, 2025). In addition to *B. japonicum* inoculation, biochar, a highly stable form of carbon produced by the pyrolysis of organic material, has gained significant attention for its role in soil enhancement. Biochar has been shown to improve soil physicochemical properties, including increased soil porosity, water retention, nutrient retention, and enhanced microbial habitat formation (Nosheen et al., 2021; Xavier et al., 2023). Adding biochar alters the soil environment by providing a stable structure that enhances microbial activity and nutrient cycling, particularly in soils with low organic matter (Dai et al., 2021; Tian et al., 2016). Moreover, biochar can adsorb and retain nutrients, particularly nitrogen, phosphorus, and potassium, thereby increasing their availability to plants (Hossain et al., 2020; Hussain et al., 2023). Higher microbial biomass, greater microbial diversity, and increased proliferation of beneficial bacteria, such as those involved in nitrogen cycling, have been associated with biochar’s effects on soil microbial communities (Abd-Alla et al., 2023; Debela Regasa and Wogi, 2020; Xu et al., 2023b).

While the individual benefits of *B. japonicum* inoculation and biochar amendment have been well-documented, several researchers have also reported the synergistic effects of these two amendments (Macil, 2018; Meena et al., 2018). It is hypothesized that biochar could provide a conducive microenvironment for *B. japonicum* bacteria, protecting them from environmental stresses such as drought, high temperatures, and pH fluctuations, thus improving their survival and nitrogen fixation activity (Ali et al., 2025; dos Santos Sousa et al., 2022; Shabir et al., 2024). This biochar, added to soil, can increase soil surface area and porosity and decrease bulk density, thus improving hydraulic conductivity and water retention capacity, soil fertility, nutrient availability, and biological properties (Sun et al., 2020). Furthermore, biochar’s capacity to enhance nutrient cycling could further stimulate the performance of *B. japonicum*, increasing the overall efficacy of biological nitrogen fixation in the soil (Lin et al., 2023; Luo et al., 2023; Ma et al., 2019). Preliminary studies suggested that combining biochar with *B. japonicum* inoculation could lead to enhanced soil microbial diversity, improved nutrient availability, and ultimately, increased crop productivity (Huang et al., 2021; Ren et al., 2020; Wu and Yan, 2024).

Given the urgent need for sustainable agricultural practices to address soil degradation and rising crop demands, this study provides essential insights into the combined use of biological amendments to improve soil fertility, sustain long-term agricultural productivity, and enhance agroecosystem resilience in a changing climate. Although numerous studies have reported the individual effects of biochar or *B. japonicum* on improving soil fertility and crop productivity, further elaboration, and a comprehensive understanding of their combined or synergistic effects on soil physicochemical properties, microbial community dynamics, and nutrient cycling under field conditions is needed. Most previous research has focused primarily on greenhouse or pot experiments, providing limited insight into how biochar influences the survival, colonization, and nitrogen fixation efficiency of *B. japonicum* in real agricultural environments. However, the interactive effects of biochar on the survival and nitrogen-fixing efficiency of *B. japonicum* within the complex microbial community of a field soil remain poorly understood, particularly how this interaction reshapes the entire soil microbiome to favor nutrient cycling. Therefore, the present study was designed to address these research gaps by investigating the integrated effects of biochar and *B. japonicum* inoculation on soil properties, microbial diversity, and soybean yield under field conditions.

This study aimed to investigate the combined effects of *B. japonicum* inoculation and biochar amendment on soil physicochemical properties, microbial diversity, and soybean yield under field conditions in Jilin Province, China. The specific objectives of this study were to: (1) assess the effects of *B. japonicum* inoculation and biochar on soil pH, total nitrogen (TN), available phosphorus (AP), potassium (AK), and soil organic matter (SOM); (2) examine the impact of *Rhizobium*-biochar amendments on the dynamics of soil nutrients throughout key soybean growth stages; (3) characterize changes in soil microbial community composition, particularly focusing on the diversity of nitrogen-cycling bacteria and mycorrhizal fungi; and (4) evaluate the functional potential of soil microbial communities, with an emphasis on nitrogen fixation and nutrients turnover.

## 2 Materials and methods

### 2.1 Field experimental site and conditions

**Figure 1:**
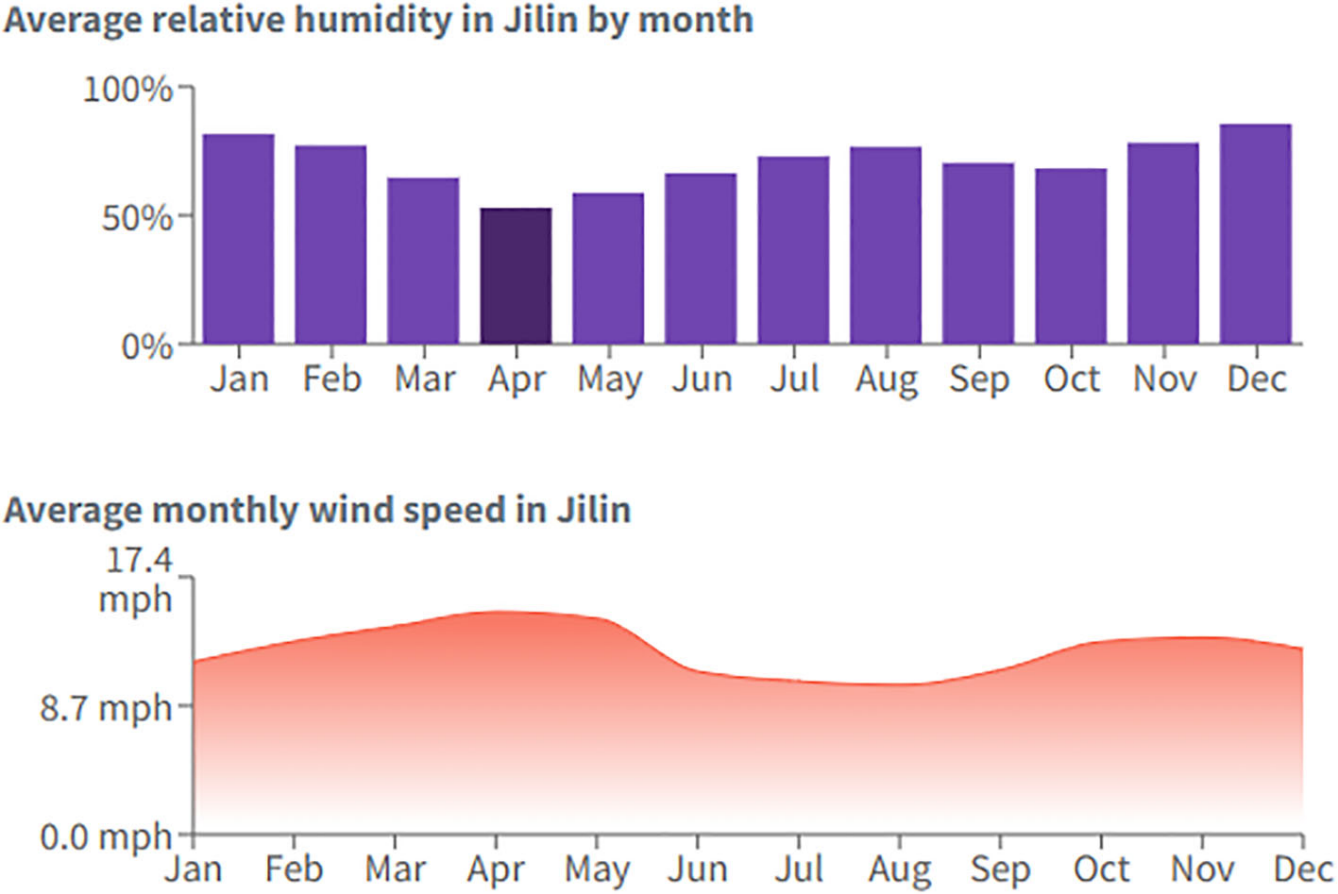
Average relative humidity and monthly wind speed at the experimental site.

**Figure 2:**
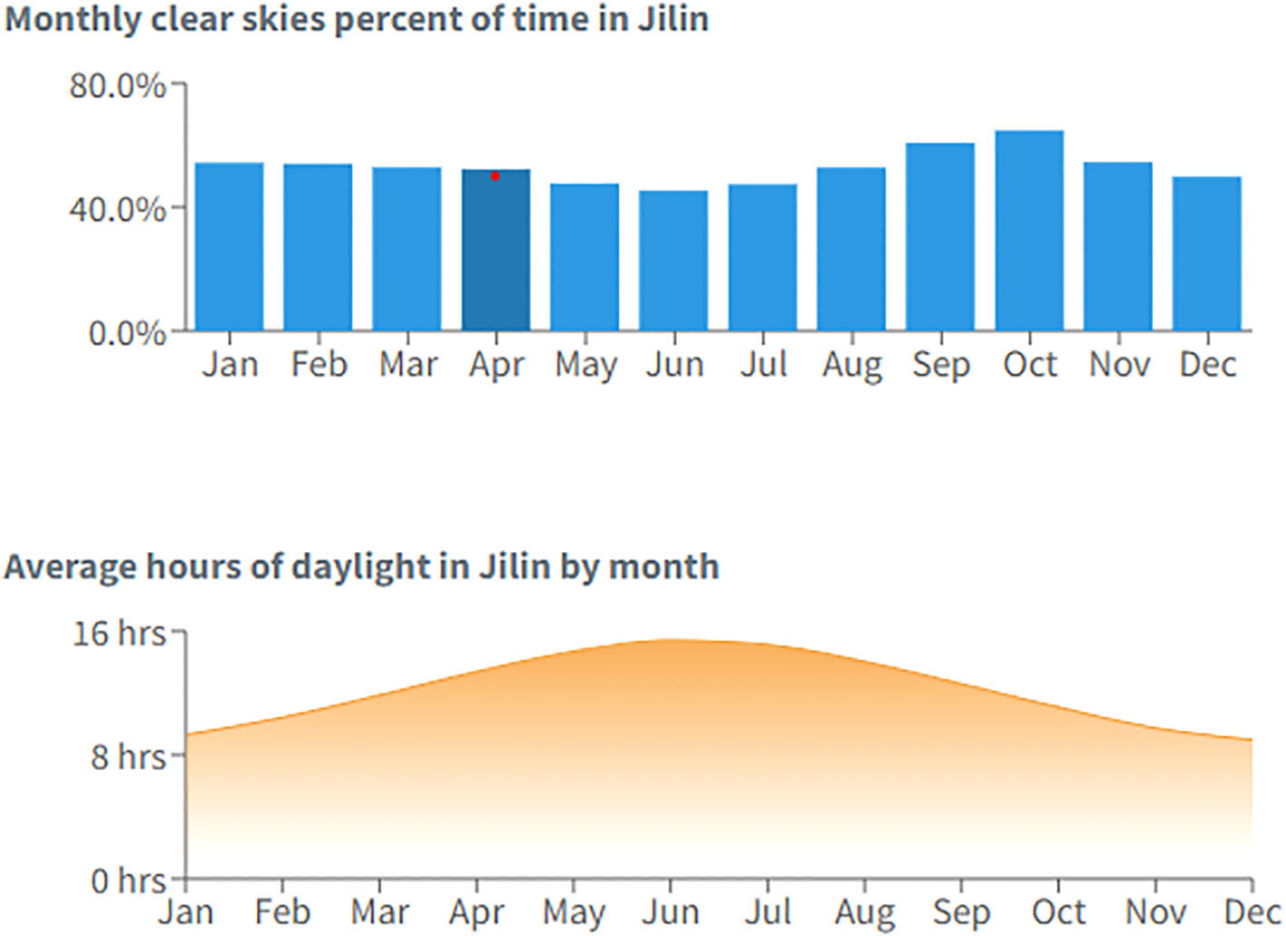
Monthly clear skies and average hours of daylight at the experimental site.

The field experimental site was located in Jilin Province, China (latitude 45.6°N, longitude 122.86°E) at an altitude of approximately 200 meters. The site experiences a temperate continental monsoon climate, characterized by hot summers and cold winters. The average annual temperature is 25.6°C, with 400 mm of annual precipitation. The average relative humidity condition, monthly wind speed, and average hours of daylight are given in **Figures 1** and **2** (source: https://wanderlog.com/weather/1065/4/jilin-weather). The soil at the site is classified as black soil, a fertile soil type commonly found in this region. The average soil pH is 5.77, indicating moderately acidic conditions. Initial nutrient concentrations in the upper 0–20 cm soil layer were: soil organic matter (SOM) @ 28.05 g·kg^−1^, available phosphorus (AP) @ 36.33 mg·kg^−1^, available potassium (AK) @ 113.64 mg·kg^−1^, and available nitrogen (AN) @ 142.45 mg·kg^−1^.

#### 2.1.1 Field experimental design and sample collections

The experiment employed a Randomized Complete Block Design (RCBD) with four treatments and three replicates per treatment. The treatments were as follows:

CK (Control)
CK2 (Biochar)
RH (*B. japonicum*)
RHB (Biochar-based *B. japonicum*)

This experimental design was intended to assess the impact of biochar and *B. japonicum* amendments on soil physicochemical properties and microbial communities. Soybean (Jiyu 47) was sown on April 29, 2024, adhering to conventional agronomic practices. Land preparation included plowing, harrowing, and ridging to ensure proper soil aeration and seedbed preparation. Sowing was carried out using a sowing machine to ensure accurate seed placement, with 50 cm row spacing and 20 cm inter-row spacing. Two seeds per hole were sown to maintain uniform plant density and minimize potential seedling loss. Each experimental plot measured 5 m × 5 m and had clearly marked boundaries to prevent cross-contamination between treatments. The biochar was derived from rice husk and pyrolyzed at 500°C. Its main properties were pH 8.7, surface area 220 m²/g, and carbon content 68.3%. The *Rhizobium* strain used was *Bradyrhizobium japonicum*, obtained from the laboratory culture collection. Biochar was applied at a rate of 300 kg per mu (4500 kg/ha), while *B. Japonicum* was applied twice during the crop’s developmental stages. The *B. japonicum* used in this study was obtained from the Agricultural Resources and Environment laboratory culture collection. The inoculum was prepared by growing the culture in Yeast Extract Mannitol (YEM) broth at 28 ± 2°C for 48 hours under continuous shaking (150 rpm). The bacterial suspension was adjusted to a density of approximately 1×10^8^ CFU mL^−1^ using serial dilution and viable plate count methods. A total of 5 L of inoculum per plot was applied immediately after ridging to promote early root colonization. Observations were recorded at the flowering, seed-filling, and harvest stages. The crop was harvested in October.

Soil samples were collected repeatedly from the same treatment plots at three growth stages (flowering (FS), seed-filling (SFS), and harvest (HS)). At each stage, five soil cores (0–20 cm depth) were taken randomly within each plot and homogenized to form one composite sample per plot. At each stage, soil samples were processed as follows: air-dried for nutrient analysis, stored at -20°C for ammonium (NH_4_⁺-N) and nitrate (NO₃⁻-N) nitrogen determinations. The soil was stored at -80°C for high-throughput sequencing to investigate microbial community structures in root-associated soil at the flowering stage.

#### 2.1.2 Measuring soil physicochemical properties

Soil properties were analyzed following established methodologies to ensure accurate measurement of key nutrients and characteristics. Soil pH was determined using the potentiometric method with a soil-to-water Ratio of 1:2.5. Available Nitrogen (AN) content was quantified using the alkali diffusion method. Available Phosphorus (AP) was assessed through sodium bicarbonate extraction followed by molybdenum-antimony colorimetry. Available Potassium (AK) levels were determined by ammonium acetate extraction followed by flame photometry. Soil Organic Matter (SOM) was measured by the potassium dichromate oxidation method with external heating. Additionally, ammoniacal nitrogen (NH4+-N) content was analyzed using indophenol blue colorimetry (Tian et al., 2024). Soil nitric nitrogen (NO₃⁻-N) content was determined using double-wavelength ultraviolet spectrophotometry, following established analytical procedures (Zang et al., 2025).

#### 2.1.3 DNA extraction, PCR amplification, and high-throughput sequencing

Total soil DNA was extracted using the OMEGA Soil DNA Kit (D5635-02) (Omega Bio-Tek, Norcross, GA, USA) according to the manufacturer’s instructions. The 16S rRNA V3-V4 region for bacterial and the ITS1 region for fungal communities were amplified using the primers 341F/806R and ITS3/ITS4, respectively. PCR amplification conditions followed protocols from the relevant literature for both bacterial and fungal targets. After sequencing, the data were processed using DADA2 in QIIME2 (2019.4) for denoising and sequence clustering.

#### 2.1.4 Method for data statistics and analysis

The assumptions of normality and homogeneity of variance were checked using the Shapiro-Wilk test and Levene’s test, respectively. The Shapiro-Wilk test indicated that the data followed a normal distribution (*p*> 0.05), and Levene’s test confirmed homogeneity of variance (*p*> 0.05). Based on these results, a One-Way ANOVA followed by Tukey’s HSD test was used to determine significant differences in soil and microbial parameters, and Origin Software was used for graphical presentation.

For microbial diversity analysis, alpha and beta diversity were assessed using Principal Coordinate Analysis (PCA) on the Genes Cloud platform (https://www.genescloud.cn). Microbial community data were processed using R (version 4.4.1). Non-metric multidimensional scaling (NMDS). Data wrangling and cleaning were carried out using the tidyverse suite, including dplyr and tidyr. Microbial ecology analyses, including diversity indices, ordination, and functional prediction, were conducted using the microeco package. FAPROTAX was applied to infer functional potential based on 16S rRNA taxonomy. Functional predictions for ecological roles such as nitrogen cycling and decomposition were conducted using FAPROTAX, with visualizations created in ggplot2.

## 3 Results

### 3.1 Effect of *B. japonicum* and biochar on soil parameters

**Figure 3:**
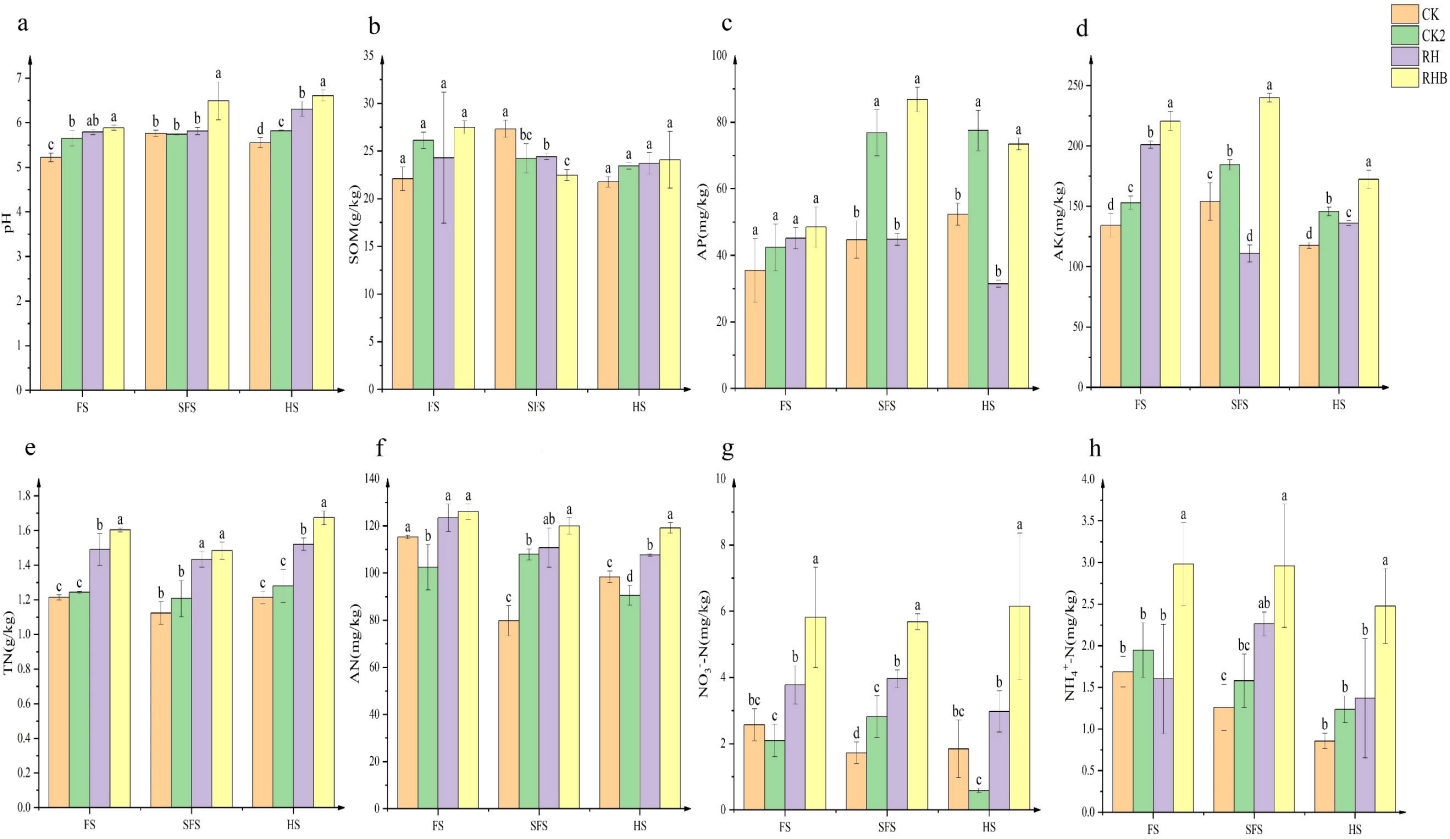
Variation in soil chemical properties across different treatment groups (CK, CK2, RH, RHB) under three experimental conditions (FS, SFS, HS). The graph illustrates the mean values of soil pH **(a)**, soil organic matter (SOM) **(b)**, available phosphorus (AP) **(c)**, available potassium (AK) **(d)**, total nitrogen (TN) **(e)**, available nitrogen (AN) **(f)**, nitrate nitrogen (NO₃⁻) **(g)**, and ammoniacal nitrogen (NH_4_⁺) **(h)** for each treatment group at the three experimental stages: FS (flowering stage), SFS (seed-filling stage), and HS (harvesting stage). Different letters above the bars indicate statistically significant differences between treatment groups within each experimental condition, as determined by *post hoc* analysis (p < 0.05). CK (Control), CK2 (Biochar), RH (*B. japonicum*), RHB (*B. japonicum* with Biochar).

RHB consistently demonstrated the highest levels of soil pH, soil organic matter (SOM), available phosphorus (AP), available potassium (AK), total nitrogen (TN), available nitrogen (AN), nitrate nitrogen (NO₃⁻-N), and ammoniacal nitrogen (NH_4_⁺-N) across all growth stages, significantly exceeding CK, which exhibited the lowest levels. At the seed filling stage, CK exhibited the highest SOM, while RHB recorded the lowest. Available phosphorus was notably higher in RHB and CK2 than in CK and RH at both the seed-filling and harvesting stages (**Figure 3**). The transient decrease in SOM in the RHB treatment at the SFS could be attributed to accelerated microbial mineralization of organic matter, driven by improved soil conditions, a phenomenon warranting further investigation.

### 3.2 Yield and correlation with soil parameters

**Figure 4:**
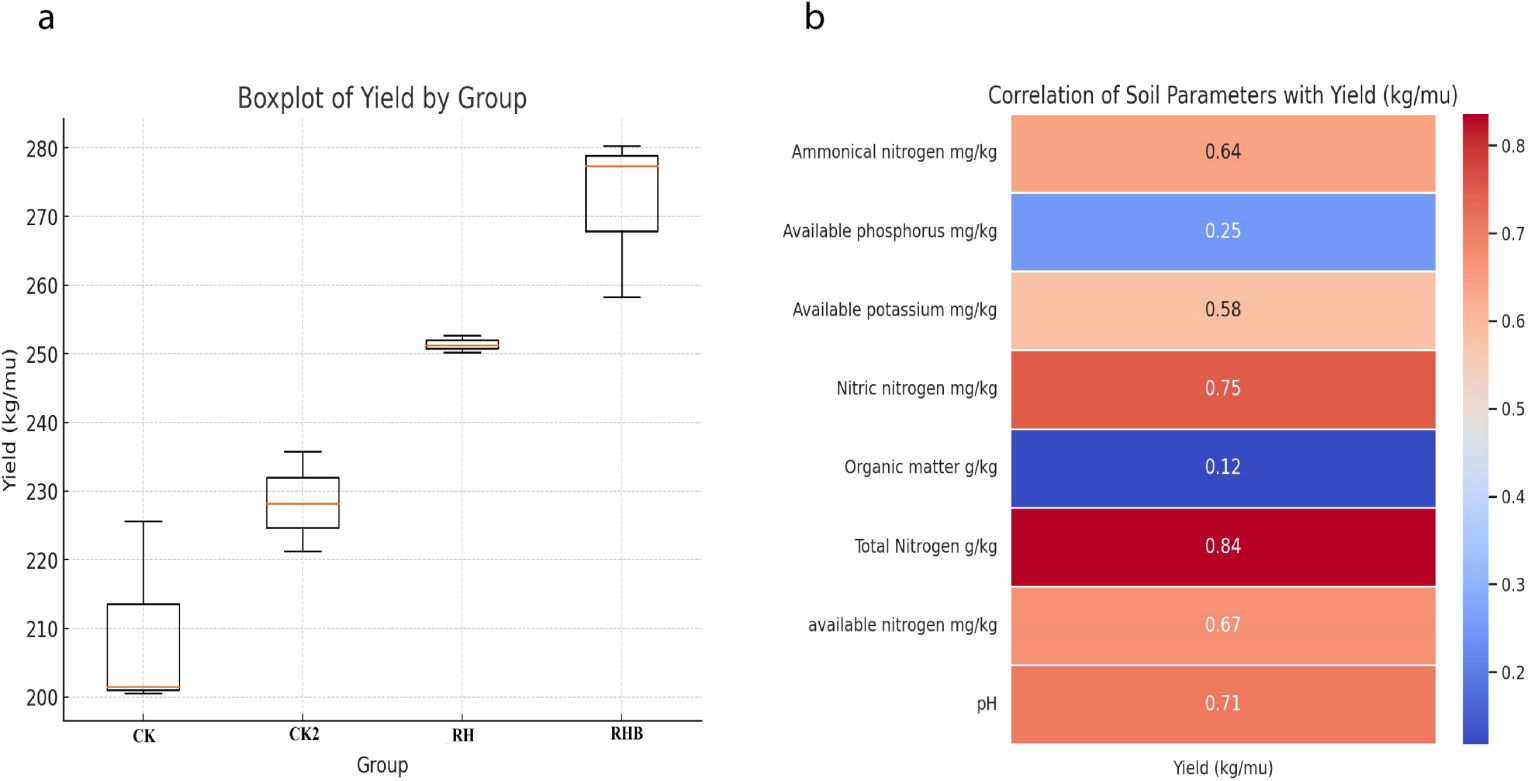
Yield and soil parameter correlations across treatments (CK, CK2, RH, RHB). **(a)** Boxplot illustrating the highest yield (kg/mu) in RHB. **(b)** Correlation analysis showing a strong positive correlation between yield and Total Nitrogen (*r* = 0.84), Nitrate Nitrogen (NO₃⁻-N) (*r* = 0.75), and Available Nitrogen (AN) (*r* = 0.67). Statistical significance was determined by *post-hoc* analysis (*p* < 0.05). CK (Control), CK2 (Biochar only), RH (*B. japonicum*), RHB (*B. japonicum* with Biochar).

The yield showed significant differences across treatments, with RHB achieving the highest yield of 250 kg/mu (3798 kg/ha) and CK the lowest at 210 kg/mu (3158 kg/ha), as evident in **Figure 4**. CK2 and RH showed intermediate yields, with slight overlap, suggesting a positive effect of the RHB treatment. Yield was strongly positively correlated with total nitrogen (TN) (r = 0.84), nitrate nitrogen (NO₃⁻-N) (*r* = 0.75), and available nitrogen (AN) (*r* = 0.67), while moderate positive correlations were observed with ammoniacal nitrogen (NH_4_⁺-N) (*r* = 0.64), available potassium (AK) (*r* = 0.58), and soil pH (*r* = 0.71). In contrast, organic matter (SOM) (*r* = 0.12) and available phosphorus (AP) (*r* = 0.25) showed weak correlations with yield.

### 3.3 Bacterial diversity and community composition

**Figure 5:**
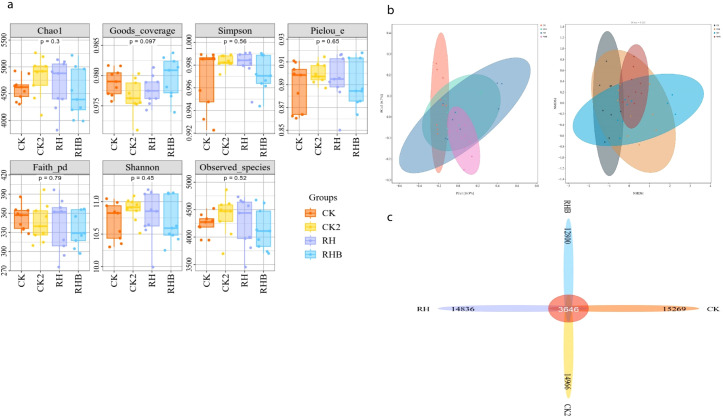
Bacterial diversity and community composition across treatments (CK, CK2, RH, RHB). **(a)** Boxplots of biodiversity indices showing no significant differences across treatments, with RHB (blue) trending towards higher diversity. **(b)** PCA and NMDS plots reveal distinct clustering of RHB, indicating a unique bacterial community structure. **(c)** Relative abundance charts show that RHB has the highest overall bacterial abundance among the treatments. CK (Control), CK2 (Biochar only), RH (*B. japonicum*), RHB (*B. japonicum* with Biochar).

There were no significant differences in bacterial biodiversity among treatments, as indicated by indices such as Chao1, Simpson, Pielou’s evenness, and Faith’s phylogenetic diversity, suggesting consistent diversity and evenness across the groups. Shannon diversity and observed species exhibited moderate variation, with RHB showing slightly higher diversity compared to CK and CK2, although these differences were not statistically significant (**Figure 5**). At the phylum level, RHB exhibited a higher relative abundance of several bacterial genera compared to CK and CK2. Although bacterial diversity (Chao1 index) did not differ significantly among treatments, the RHB treatment markedly altered bacterial composition, enriching beneficial genera such as *Pseudomonas* and *Bacillus*. Conversely, fungal richness increased significantly with RHB, suggesting that bacteria and fungi respond differently to the combined treatment, reflecting a more functionally active and balanced microbial community. These results suggest that, although slight differences were observed, the overall bacterial community structure remained relatively stable across the treatments.

### 3.4 Bacterial community composition and taxonomic shifts

**Figure 6:**
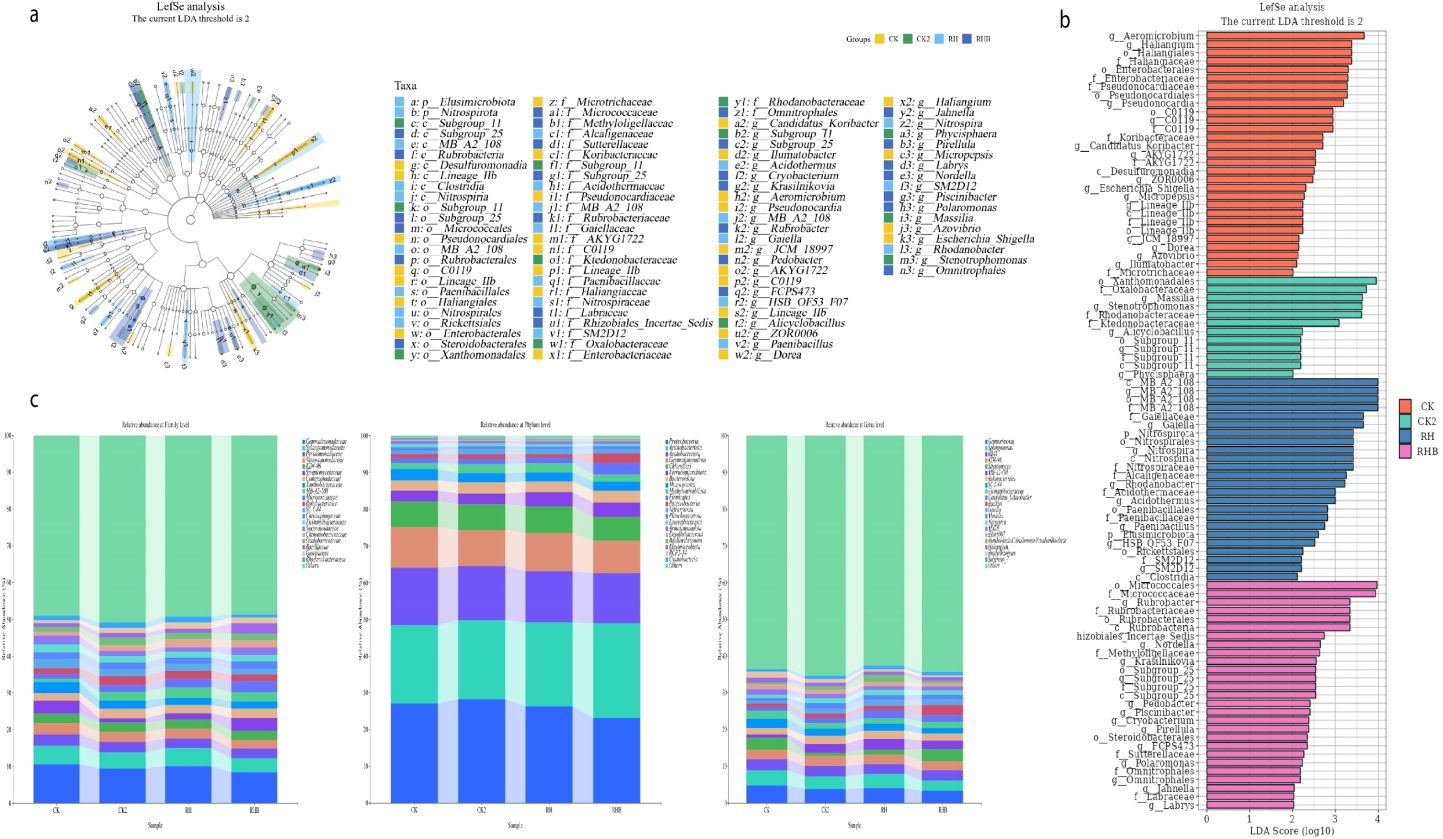
Bacterial community composition across treatments (CK, CK2, RH, RHB). **(a)** LEfSe (Linear discriminant analysis Effect Size) analysis showing taxonomic distribution of bacterial genera, with significant taxa indicated by treatment group colors. **(b)** LDA (Linear discriminant Analysis) scores highlight key discriminative taxa: RHB is enriched in Candidatus Koribacter and Pseudomonas, and CK is associated with *Micrococcaceae* and *Streptomyces*. **(c)** Stacked bar charts displaying bacterial abundance at various taxonomic levels, revealing distinct microbial profiles in RHB. CK (Control), CK2 (Biochar), RH (*B. japonicum*), RHB (*B. japonicum* with Biochar).

Notable differences in bacterial community composition were observed across treatments. RHB exhibited significantly higher abundances of Pseudomonas and Nitrosospira, whereas CK showed higher abundances of *Micrococcaceae and Streptomyces*. Taxa such as *Elusimicrobiota*, *Nitrospira*, and *Candidatus Koribacter were* particularly enriched in RHB, suggesting treatment-driven shifts in microbial community composition (**Figure 6**). These findings suggest that RHB fosters a distinct microbial profile characterized by increased abundance of beneficial bacteria involved in nutrient cycling, which may contribute to enhanced soil health.

### 3.5 Fungal community diversity and composition

**Figure 7:**
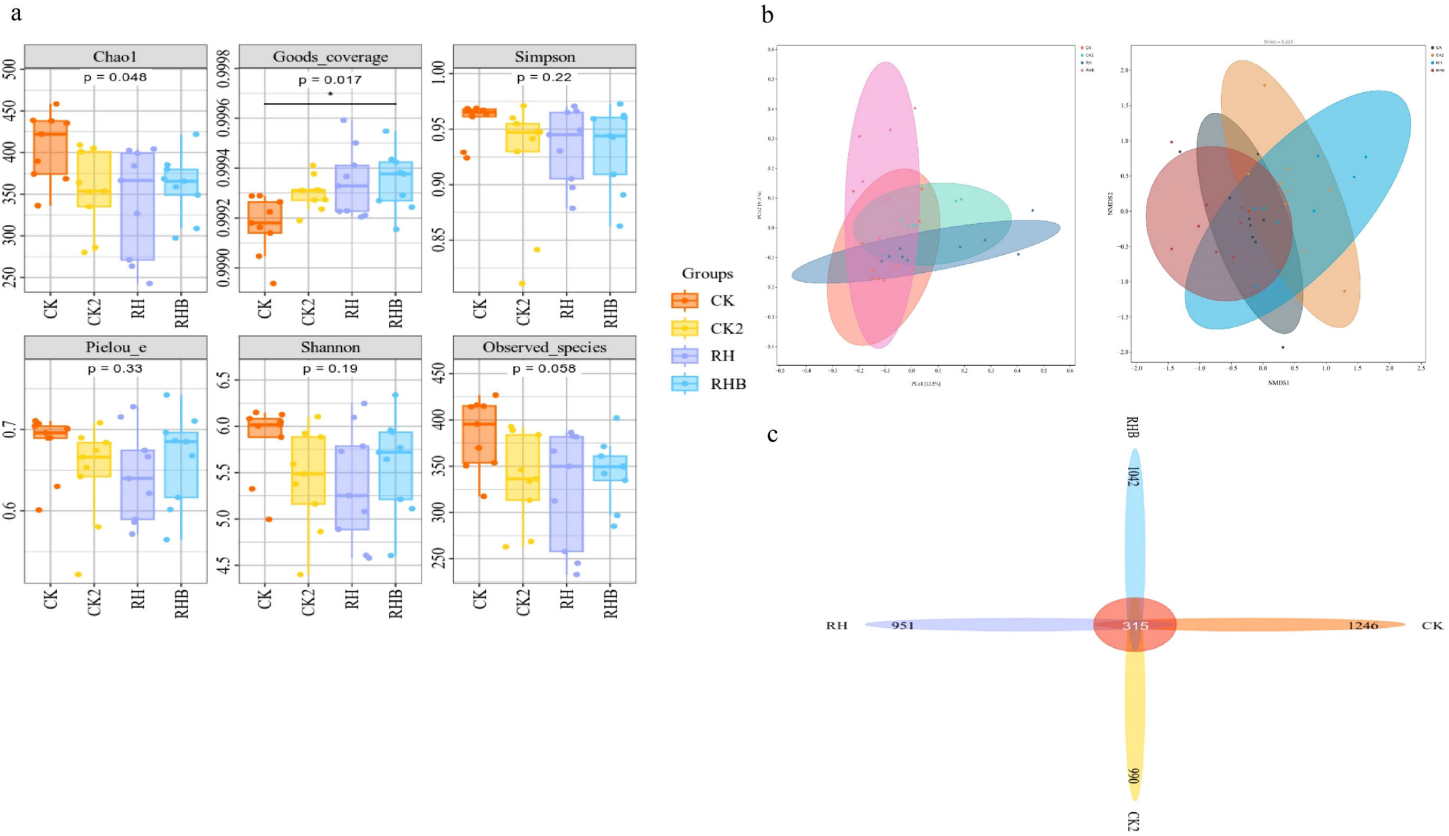
Fungal diversity and abundance across treatments (CK, CK2, RH, RHB). **(a)** Boxplots of biodiversity indices showing higher fungal richness and coverage in RHB. **(b)** PCA and NMDS plots reveal distinct clustering of RHB (blue), indicating a unique fungal community structure. **(c)** Heatmap showing higher abundance of specific fungal genera in RHB compared to other treatments. CK (Control), CK2 (Biochar), RH (*B. japonicum*), RHB (*B. japonicum* with Biochar).

Fungal diversity across the four treatment groups (CK, CK2, RH, and RHB) was assessed using biodiversity indices. RHB exhibited significant increases in richness and community coverage, as indicated by higher Chao1 (p = 0.048) and Goods coverage (p = 0.017) values compared to CK and CK2. Other indices, including Shannon diversity (p = 0.19) and Simpson’s diversity (*p* = 0.22), did not differ significantly, suggesting consistent fungal evenness and overall diversity across treatments (**Figure 7a**). At the genus level, RHB showed higher relative abundances of key genera, indicating that the treatment promotes fungal diversity and the presence of key genera involved in nutrient cycling (**Figure 7**).

### 3.6 Fungal community composition and functional profiling

**Figure 8:**
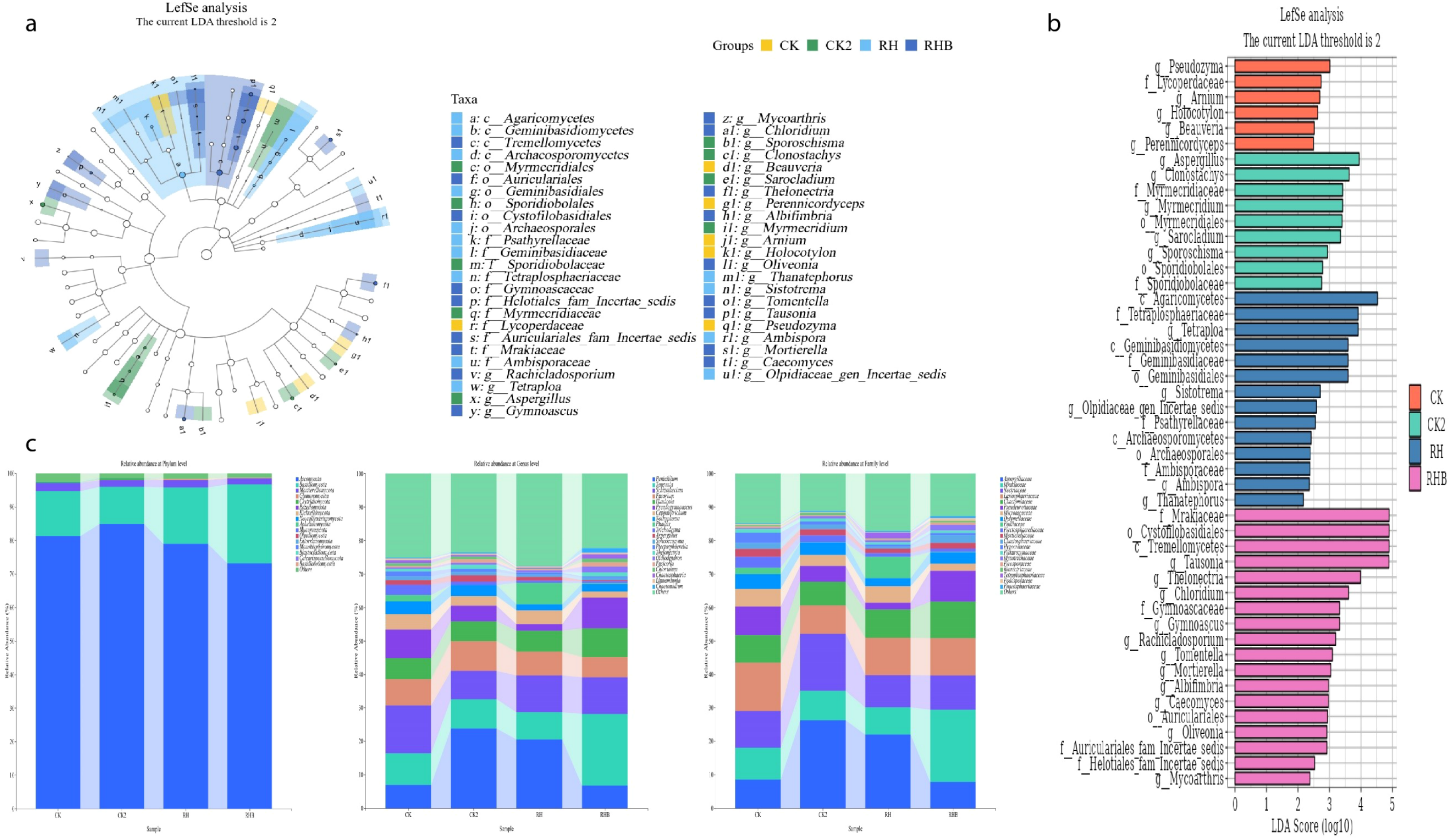
Fungal community composition and functional profiles across treatments (CK, CK2, RH, RHB). **(a)** LEfSe analysis indicates differential abundances of fungal taxa among treatments. **(b)** LDA scores highlight the dominant fungal taxa in each treatment group, with RHB (blue) showing a strong association with specific genera. **(c)** Relative abundance at the family and genus levels reveals distinct fungal profiles in RHB compared to other treatments. CK (Control), CK2 (Biochar), RH (*B. japonicum*), RHB (*B. japonicum* with Biochar).

Differential fungal community composition and functional profiles across treatment groups revealed distinct patterns in fungal taxa, as evident from **Figure 8**. *Pseudozyma, Lycomycetaceae*, and *Myrmecidales* were enriched in CK and RH, while *Beaveria, Tetraspora*, and *Ambispora* were more abundant in RHB, demonstrating that the treatments influence fungal community structure. RHB was strongly enriched in *Pseudozyma, Beaveria*, and *Myrmecidales*, while CK showed higher abundance of *Pseudozyma* and *Ambispora*, confirming that RHB promotes a distinct fungal profile (**Figure 8a**). Taxonomic distribution at the family level revealed that RHB was enriched in *Mycothrix, Tremellomycetes*, and *Aspergillus*, whereas CK had a greater abundance of *Auriculariaceae* and *Pseudozyma*. At the genus level, RHB was dominated by *Myrmecidales*, *Beaveria*, and *Ambispora*, further emphasizing the distinct fungal community structure shaped by RHB treatment.

### 3.7 Functional profiling of bacterial and fungal communities

**Figure 9:**
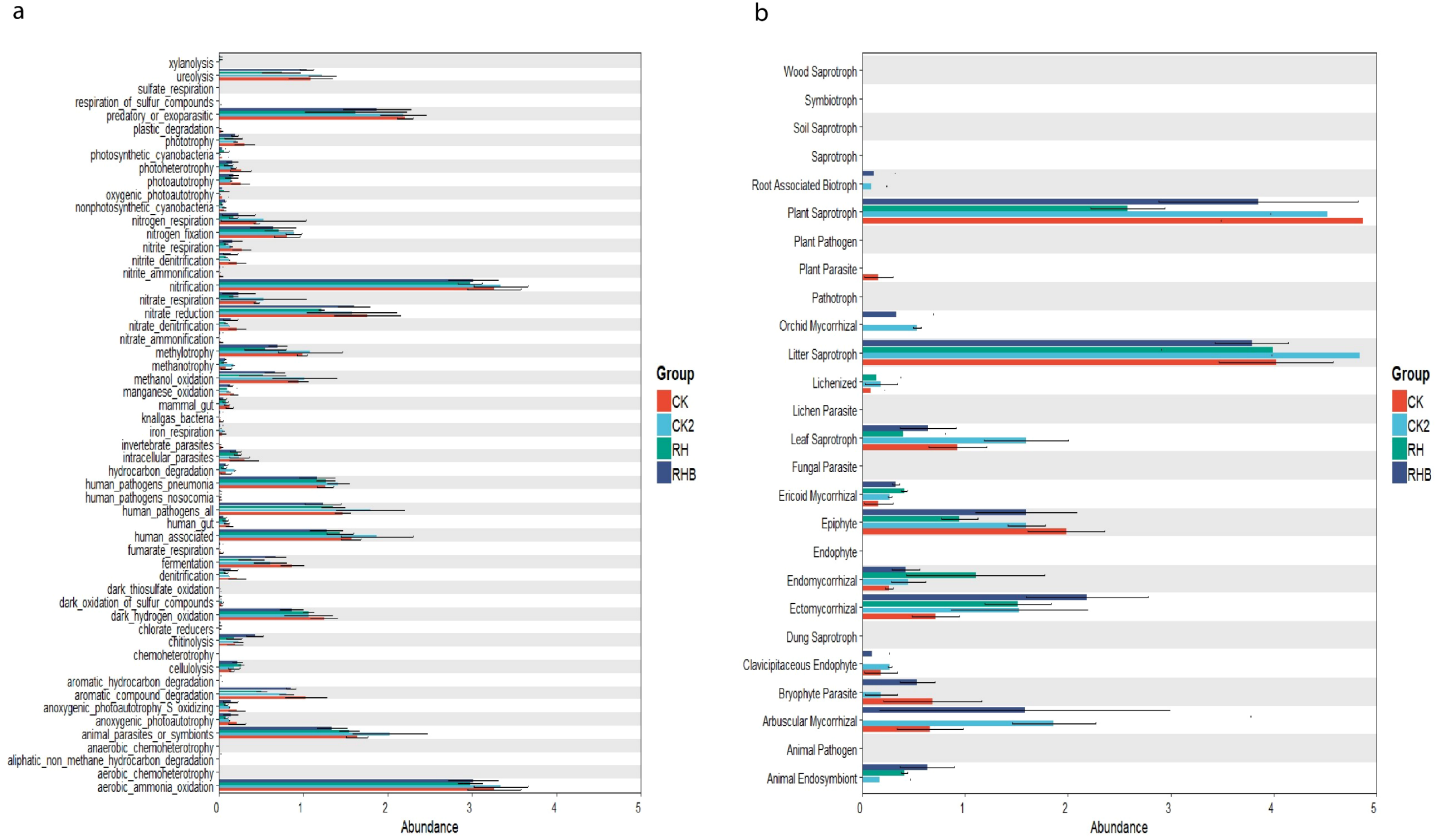
**(a)** Functional Profiling of different Bacterial Communities, such as ammonia oxidation and nitrification. **(b)** Functional Profiling of Fungal Communities, such as saprotrophs and parasites. RHB shows increased bacterial abundance in nitrogen cycling and oxidative processes. Fungal communities in RHB exhibit a higher abundance of mycorrhizal and root-associated biotrophs, while CK shows a greater presence of plant pathogens and fungal parasites. Relative abundance data are presented in bar charts, and statistical significance is determined by appropriate analyses. CK (Control), CK2 (Biochar), RH (*B. japonicum*), RHB (*B. japonicum* with Biochar).

Bacterial functional groups in RHB exhibited higher abundances of those involved in nitrogen cycling, including nitrite denitrification and methanotrophy, compared to CK and CK2. In contrast, CK showed a higher abundance of groups related to sulfate respiration. Fungal functional groups in RHB were enriched in mycorrhizal fungi, saprotrophs, and root-associated biotrophs, suggesting enhanced plant-fungal symbioses and organic matter decomposition. Conversely, CK was dominated by plant pathogens and fungal parasites, indicating a shift towards potentially harmful microbial taxa. CK2 and RH exhibited more balanced fungal communities, with moderate levels of beneficial fungi (**Figure 9**).

### 3.8 Correlation and RDA of key bacterial and fungal taxa with soil parameters and yield

**Figure 10:**
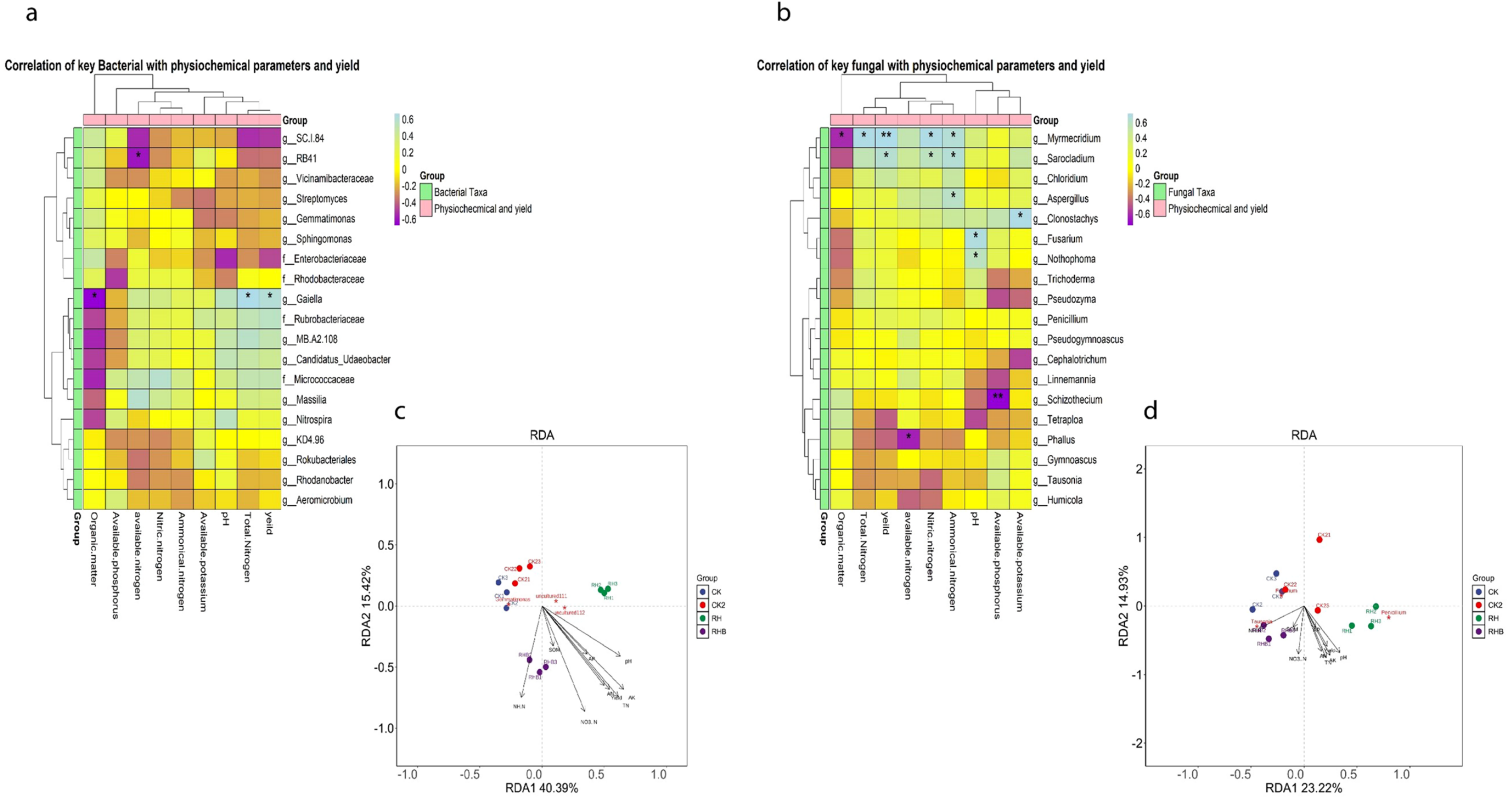
Correlation of Microbial Communities with Soil Parameters and Yield. **(a)** Heatmap of bacterial genera’ correlations with soil traits and yield; significant correlations marked with (*). **(b)** Heatmap of fungal genera correlations with soil traits and yield; significant once marked (*). RHB (blue) shows the strongest links. **(c)** RDA plot for bacteria highlights soil-driven distribution across treatments. **(d)** RDA plot for fungi shows distinct RHB clustering based on soil and yield. Highly significant once marked (**).

Strong correlations were observed between bacterial genera, including Streptomyces, Sphingomonas, and Rhodobacteraceae, and key soil parameters, total nitrogen, ammoniacal nitrogen, available phosphorus, and crop yield under RHB treatment (**Figure 10**). Similarly, fungal genera such as *Myrmecidrium*, *Sarocladium*, and *Aspergillus* were positively correlated with available phosphorus and organic matter, while *Fusarium* and *Nothophoma* were associated with ammoniacal nitrogen and available nitrogen. RHB significantly influenced microbial composition, with distinct clustering observed in both bacterial and fungal communities, emphasizing its substantial impact on soil nutrient dynamics and crop productivity.

## 4 Discussion

This study demonstrates that integrating *B. japonicum* inoculation with biochar (RHB) significantly enhanced soil fertility and microbial diversity compared with the individual treatments of biochar alone (CK2) and *B. japonicum* (RH). The RHB treatment led to substantial improvements in key soil properties, including total nitrogen (TN), available nitrogen (AN), nitrate nitrogen (NO₃⁻-N), soil organic matter (SOM), and soil pH. These findings substantiate the hypothesis that biochar serves not only as a physical soil amendment but also as a facilitator of nitrogen and other nutrient bioavailability (Rasse et al., 2022; Xu et al., 2023a). The porous structure of biochar creates a stable microenvironment for *B. japonicum*, promoting its survival and activity (Shabir et al., 2024, Shabir et al., 2023). The ability of biochar to adsorb and retain nitrogen is well-documented (Rao et al., 2024). Our results align with these studies by demonstrating a significant increase in nitrogen availability and soil fertility under RHB. Additionally, the RHB treatment promoted a more stable and diverse microbial community, which is crucial for nutrient cycling and soil health.

The microbial community composition in RHB was distinctly different from that in CK, CK2, and RH treatments. RHB showed increased abundances of beneficial bacterial genera involved in nitrogen cycling and oxidative processes, such as *Nitrosospira* and *Pseudomonas*, alongside a higher presence of mycorrhizal fungi and root-associated biotrophs. These microorganisms are pivotal in nitrogen fixation, organic matter decomposition, and phosphorus uptake (Celi et al., 2022; Tian et al., 2021). The increased presence of these microbes in RHB likely contributed to improved nutrient availability, thereby fostering greater soybean yield and plant productivity. This is consistent with prior studies showing that *B. japonicum* promotes plant growth through efficient nitrogen fixation (Goyal et al., 2021; Jaiswal et al., 2021), while biochar enhances microbial communities by improving soil structure and facilitating nutrient retention (Wang et al., 2017; Zhang et al., 2023).

The results also indicate that RHB promotes not only nutrient cycling but also enhances microbial functional diversity, which is integral to maintaining soil health and supporting crop productivity. The presence of mycorrhizal fungi and root-associated biotrophs in RHB suggested enhanced plant-microbe symbiosis, a key factor in improving soil nutrient dynamics and increasing stress resilience. In particular, mycorrhizal fungi enhance phosphorus availability and water retention in soil, and their increased abundance in RHB may contribute to the observed improvements in soybean yield. The shift in the fungal community structure in RHB, particularly toward beneficial taxa such as mycorrhizal fungi and root-associated biotrophs, further supports the positive effects of this treatment on soil health and plant productivity.

In contrast, although some improvements in soil pH and available phosphorus were evident in the CK and CK2 treatments, they did not exhibit similar improvements in nitrogen cycling or microbial diversity. The CK2 treatment showed slight increases in soil pH and available phosphorus, but it did not enhance microbial activity as observed for RHB. These improvements did not translate into significant increases in microbial diversity or soybean yield. This suggested that although biochar alone can improve soil structure and nutrient retention, it does not have the same capacity to enhance nitrogen cycling or microbial functional diversity as when combined with *B. japonicum.* These findings support the notion that *B. japonicum* inoculation is essential to fully capitalize on biochar’s benefits by facilitating biological nitrogen fixation and promoting a more productive microbial community.

The soybean yield was significantly higher in RHB than in the other treatments, and the strongest positive correlations were observed between yield and nitrogen-related parameters, including total nitrogen, available nitrogen, and nitrate nitrogen. These results highlight the critical role of nitrogen in legume productivity, especially in soils where nitrogen is a limiting factor. The observed improvements in yield with RHB are likely due to the combined effects of enhanced nitrogen availability and the stimulation of beneficial microbial communities, which facilitate more efficient nutrient uptake and promote soil health.

While the immediate benefits of RHB on soil fertility and soybean yield are evident, further investigation into the long-term sustainability of this combined treatment is essential. Future research should focus on assessing the long-term impacts of *B. japonicum* and biochar on soil microbial stability, ecosystem services, and nutrient cycling under various environmental conditions. Specifically, studies evaluating the effects of RHB on carbon sequestration, soil resilience, and ecosystem functions over multiple growing seasons would provide critical insights into the sustainability of this approach. Additionally, understanding the underlying mechanisms of microbial interactions, particularly how biochar and *B. japonicum* work synergistically to enhance soil microbial diversity, will be essential for optimizing this combined treatment for broader agricultural applications.

## 5 Conclusion

The combined application of *B. japonicum* and biochar improved soil fertility, enhanced microbial diversity, and increased soybean yield compared with their individual use. More importantly, this integration highlights a novel, synergistic strategy where biochar provides a stable habitat that sustains microbial inoculants, while *B. japonicum* contributes to biological nitrogen fixation and nutrient cycling. From a practical standpoint, this approach provides farmers with a low-cost, environmentally friendly alternative to chemical fertilizers, thereby reducing input costs and mitigating soil degradation. Beyond yield benefits, it also supports soil carbon sequestration, promotes beneficial microbial communities, and strengthens agroecosystem resilience, key requirements for sustainable agriculture in the face of climate change.

Future research should focus on long-term field trials across diverse soils and climatic conditions to confirm broader applicability and evaluate ecosystem-level impacts, including carbon storage and soil resilience. By integrating microbial biotechnology with soil amendments, this study offers a scalable and sustainable approach to enhance crop productivity while conserving natural resources.

## Data Availability

The original contributions presented in the study are publicly available. This data can be found here: https://ngdc.cncb.ac.cn/, accession number CRA034741.
